# Obesity and Blood Pressure in 17-Year-Old Offspring of Mothers with Gestational Diabetes: Insights from the Jerusalem Perinatal Study

**DOI:** 10.1155/2011/906154

**Published:** 2011-07-24

**Authors:** Meytal Avgil Tsadok, Yechiel Friedlander, Ora Paltiel, Orly Manor, Vardiella Meiner, Hagit Hochner, Yael Sagy, Nir Sharon, Shoshanah Yazdgerdi, David Siscovick, Uriel Elchalal

**Affiliations:** ^1^Epidemiology Unit, Hebrew University Braun School of Public Health, P.O. Box 12272, Jerusalem 91120, Israel; ^2^Department of Human Genetics, Hadassah-Hebrew University Medical Center, Jerusalem 91120, Israel; ^3^Department of Medicine and Epidemiology, University of Washington, Seattle, WA 98101, USA; ^4^Obstetric and Gynecology Department, Hadassah-Hebrew University Medical Center, Jerusalem 91120, Israel

## Abstract

*Objective*. Gestational diabetes mellitus (GDM) influences fetal development and offspring's metabolic risk. We evaluated this association in 17-year-old offspring adjusting for birth weight (BW) and prepregnancy maternal BMI (mBMI). *Study Design*. The JPS birth cohort contains extensive data on 92,408 births from 1964 to 1976. Offspring's BMI and blood pressure (BP) were obtained from military records. For a subcohort born between 1974 and 1976, prepregnancy mBMI was available. Offspring were classified as born to mothers with GDM (*n* = 293) or born to mothers without recorded GDM (*n* = 59,499). *Results*. GDM offspring had higher mean BMI and systolic and diastolic BP compared to no-recorded-GDM offspring. After adjusting for BW, GDM remained significantly associated with offspring BMI and diastolic BP (*β* = 1.169 and 1.520, resp.). In the subcohort, when prepregnancy mBMI was entered to the models, it markedly attenuated the associations with GDM. *Conclusions*. Maternal characteristics have long-term effects on cardiometabolic outcomes of their offspring aged 17 years.

## 1. Introduction

Fetal development is regulated by maternal and fetal characteristics that influence the intrauterine environment. The adaptation of the fetus to intrauterine conditions results in fetal “programming” which may also determine the metabolic “fate” of the fetus later in life [1]. 

Gestational diabetes mellitus (GDM) is defined as carbohydrate intolerance with different degrees of severity, first diagnosed during pregnancy [2]. It is the most common metabolic complication during pregnancy and is observed in 3%–5% of all pregnant women. 

Intrauterine hyperglycaemic conditions influence fetal metabolism and in turn may influence later life morbidity. The short-term consequences of GDM for the offspring are high birth weight [3] and associated perinatal complications such as shoulder dystocia, hypoglycaemia, hyperbilirubinemia, hypocalcemia, and polycythemia [4]. The intermediate-term consequences include impaired insulin resistance, obesity, and type 2 diabetes during childhood [5–7]. Few studies reported an association between maternal GDM and elevated blood pressure later in offspring life [8, 9], but most of the studies that investigated the long-term consequences of GDM on offspring have not reported analyses of blood pressure measurements. 

There is some evidence that neonatal birth weight [10], maternal weight gain during pregnancy [11], and prepregnancy maternal BMI [12, 13] (mBMI) are also important factors predicting long-term morbidity of offspring; however, the impact of these factors on the associations of GDM and the long-term health of the offspring remains unclear. 

In a previous report [14] on a subset of the Jerusalem perinatal study (JPS) (offspring born in Jerusalem between 1974 and 1976), there was evidence of an increased risk of adolescent overweight, defined as a body mass index (BMI) >90th percentile, among offspring born to diabetic mothers. However, this report included only univariate correlations and did not analyze the data using multivariate models, taking into account various important factors such as prepregnancy mBMI. 

In this study, we aimed to assess the long-term implications of maternal GDM on adolescent BMI and blood pressure, among JPS offspring at age 17, after taking into account different characteristics, such as offspring birth weight and prepregnancy mBMI.

## 2. Methods

### 2.1. Population

#### 2.1.1. Whole Cohort

The Jerusalem perinatal study (JPS) is a population-based cohort that includes all births occurred in western Jerusalem and its surroundings between 1964 and 1976. Detailed information on data collection has been previously described [15]. In brief, demographic, socioeconomic, and clinical data (medical conditions of the mother including GDM status during all pregnancies from 1964 to 76), and offspring birth weight were collected. The information was extracted either from birth certificates or maternity ward logbooks at the time of birth. 

Data on maternal conditions, obstetric complications, and interventions during labor and delivery, including GDM status, were collected systematically in 92,408 deliveries. The original data collection instruments included rubrics for GDM (i.e., diabetes with onset during pregnancy) and pre-gestational diabetes (i.e., diabetes, mostly insulin-dependent diabetes, prior to the present pregnancy). In this study, we used only the definition for GDM health condition and did not included the pregestational diabetes cases. In that era, all pregnant women were screened for glycosuria at each antenatal visit, and if found positive, they would be referred for an oral glucose tolerance test.

#### 2.1.2. Subcohort

Postpartum interviews of a subset of mothers (17,003, referred to as the subcohort) who gave birth between November 1974 and the end of 1976 provided further information on medical conditions and lifestyle habits, including prepregnancy mBMI, weight gain during pregnancy, gestational age at delivery, and smoking habits. These interviews were conducted at the bedside on the first or second day postpartum by nurse-midwives and captured 98% of the births.

#### 2.1.3. Military Records

Through the Israeli Population Registry, we verified the identities of 99% of the offspring and 96.2% of the mothers in the JPS cohort using their unique identity (ID) numbers. The ID number and other identifiers enabled us to link the JPS data with military draft records. This linkage provided information regarding the weight, height, and systolic and diastolic blood pressure which were measured in 60,191 JPS singleton offspring (37,308 males and 22,883 females) at age 17.

### 2.2. Study Variables

The following variables were included in the analysis: parents' ages at birth, birth order, offspring's birth weight (continuous variables), maternal level of education (years of schooling grouped into three categories: 0–8 years, 9–12 years and ≥13 years) and socioeconomic status (SES) using a scale based on husband occupation (grouped into three categories: high, middle, and low). Maternal ethnic origin was classified according to her father's country of birth (categorized as: Israel, West Asia, North Africa and Europe/America). 

Maternal medical history included data on diabetes and pre-eclampsia in the current pregnancy or in other pregnancies (between 1964 and 1976, recorded in the JPS database). 

Information on maternal smoking during pregnancy, gestational age at delivery (calculated from the date of last menstrual period), prepregnancy mBMI, and weight gain during pregnancy was available only in the subcohort of the mothers who gave birth between 1974 and 1976. 

Anthropometric and blood pressure information measured in the offspring at age 17 were obtained from the Israel Defense Forces medical database. Details about these examinations have been described elsewhere [13]. Briefly, blood pressure was measured in the sitting position in the right arm with a Bauman sphygmomanometer with appropriate cuff size. Standing height was measured on barefooted subjects, and body weight was measured with light indoor clothing. BMI was calculated as the ratio of weight (kg) to standing height (m) squared (kg/m^2^). The examiners were blinded to the perinatal data.

The current study examined the differences in offspring BMI and blood pressure measured at age 17 among the two groups: offspring whose mothers had GDM in the index pregnancy (GDM) and offspring whose mothers did not have GDM in any of her pregnancies during the period 1964–1976 and recorded in the JPS (no-recorded-GDM). We included only singleton pregnancies in this analysis.

### 2.3. Statistical Analysis

Baseline characteristics of the study population and variables obtained at age 17 are presented for the two groups of offspring categorized by their mother's GDM status. Descriptive analysis was used to compare the BMI and blood pressure mean values for the offspring groups, and linear regression analysis was used to adjust for other characteristics in multivariate models. 

For the 1964–1976 subcohort, two sets of models were examined: (1) model 1 included the maternal GDM status as the main covariate (GDM compared to the no-recorded-GDM group) and (2) model 2 additionally adjusted for offspring birth weight. These models were also adjusted for the following covariates: maternal age at birth, ethnic origin (Israel as a reference group), level of education (0–8 years as a reference group), maternal pre-eclampsia (in one or more pregnancies between 1964 and 1976), birth order, SES (middle class as a reference group), and offspring gender.

For the subcohort of 1974–1976, we further adjusted for prepregnancy mBMI and weight gain during the pregnancy (model 3). In the 1974–1976 analysis, maternal smoking and gestational age at delivery were also included in the models as covariates. 

To account for the correlation between siblings within families, the multivariate analysis for the entire cohort (1964–1976) was repeated using a mixed linear model (SAS PROC MIXED). Both approaches provided similar findings; and therefore, the results from the linear regression models are presented. Analyses were conducted with SPSS version 14.0 (Chicago, Ill, USA) and SAS package version 9.1 (Cary, NC, USA).

## 3. Results

### 3.1. Characteristics of the Cohort

Of the 92,408 deliveries recorded in the JPS between 1964 and 1976, military induction examination information was available for 60,191 (65.1%) singleton offspring. Of these, 293 (0.5%) were born to mothers with GDM and 59,499 (98.8%) to mothers with No-recorded-GDM. 

An additional 399 (0.7%) offspring were born to mothers who had GDM not in the index pregnancy but in one of her other pregnancies during the period 1964–1976 and recorded in the JPS. These offspring were not included in the analysis. 

Of the 16,912 singleton deliveries during the 1974–1976 subcohort, military information was available for 11,412 offspring, of whom 77 offspring were born to GDM mothers, and 11,335 to no-recorded-GDM mothers. 

Table 1 describes the parental characteristics of study participants of the two groups of offspring. Both maternal and paternal ages were higher, on average, among infants exposed to GDM in utero. Mean birth weight of the GDM group (3411 ± 616 g) was higher than the no-recorded-GDM group (3301 ± 483 g  *P* < 0.001). 

Mothers in the GDM group were more educated and from higher SES status compared to mothers from the no-recorded-GDM group. In addition, mothers with GDM were more likely to have pre-eclampsia in one of their pregnancies recorded in the JPS, compared to mothers belonging to the no-recorded-GDM group.

### 3.2. Anthropometry and Blood Pressure at Age 17

Table 2 presents the gender standardized anthropometric and blood pressure values measured in offspring at age 17, classified by mother's GDM status. 

BMI, systolic and diastolic blood pressure mean values were significantly higher in GDM offspring as compared to the mean values obtained in offspring born to no-recorded-GDM mothers (*P* < 0.05 for all outcomes).

### 3.3. Predictors for BMI and Blood Pressure at Age 17

Table 3 demonstrates the association of GDM with BMI and systolic and diastolic blood pressure of the offspring measured at age 17. 

Among offspring who were born between 1964 and 1976, maternal GDM was positively and significantly associated with offspring BMI values (model 1) independent of BW (model 2). 

In the analysis of the 1974–1976 subcohort, when models were further adjusted for maternal smoking and gestational age at delivery, GDM was positively and significantly associated with offspring BMI measured at age 17 (model 1); however, when birth weight was included in the model (model 2), it attenuated the association with GDM. When prepregnancy mBMI and weight gain during pregnancy were included in the model (model 3), the association of GDM with the offspring BMI was attenuated markedly. 

When we evaluated the association of GDM with systolic blood pressure measured in offspring at age 17, no significant associations were found in the entire cohort and in the subcohort as well (model 1). When prepregnancy mBMI included in the analysis of the subcohort, it was positively associated with systolic blood pressure (model 3). 

In distinction to systolic blood pressure, GDM was significantly associated with diastolic blood pressure in the entire cohort, even after introducing birth weight to the model (model 2). In the subcohort models, there was no evidence of an association between GDM and offspring diastolic blood pressure, and prepregnancy mBMI was positively associated with diastolic blood pressure at age 17.

## 4. Comments

Our study implies that maternal characteristics pre- and during pregnancy and the intrauterine environment are important factors for future cardiometabolic conditions of their offspring. 

In the entire JPS cohort of 1964–1976, we found that the contribution of maternal GDM to offspring BMI in young adulthood is independent of neonatal birth weight. These findings are consistent with previous studies [16–18]. However, the analysis of the smaller and more detailed subcohort indicates that when prepregnancy maternal BMI is included in the model, the association of maternal GDM with offspring BMI at age 17 is considerbly reduced (Table 3). 

Our study suggested a positive association between maternal GDM and diastolic blood pressure measured in the 17-years-old offspring, in the whole cohort. Other studies, focusing on childhood blood pressure, have demonstrated higher blood pressure values in offspring of mothers with GDM [8, 9], while others have not [6]. 

Even though the association between GDM and diastolic blood pressure did not reach statistical significance in the smaller subcohort (1974–1976), the coefficient of GDM was attenuated when prepregnancy mBMI was included in the model. This implies that the association between maternal GDM and offspring diastolic blood pressure in young adulthood might as well be accounted for prepregnancy mBMI. 

There is growing evidence in the literature, that prepregnancy mBMI is a strong predictor of offspring health status later in life [6, 19–21]. Regarding GDM, other studies concluded that maternal obesity and diabetes are independent risk factors for adverse short-term perinatal outcomes [22–24]. Our study suggests that the effect of maternal GDM on cardiometabolic outcomes in their 17-year-old offspring is not independent of prepregnancy mBMI. 

This study has potential limitations that should be considered. During the JPS data collection period (1964–1976), screening for GDM was not routine in Israel and the prevalence of GDM in the cohort was lower (0.5%) than the current reported prevalence of 3%–5%, possibly due to differences both in the diagnosis of GDM, and in the study population, as 85% of the mothers had a pre pregnancy BMI less than 25 at that time. In addition, we do not have information regarding GDM severity or mode of treatments of the mothers. We assume that the more severe cases of GDM were ascertained and diagnosed at that time; therefore, our results may represent the associations for the offspring of a group of mothers with a more severe form of GDM rather than the GDM detected today by screening during pregnancy. Another limitation is that military induction examination information was available for only 65.1% of the offspring included in the JPS cohort. In the Israeli Defense Force, military service is compulsory for all Jewish males and females, but female subjects are less commonly recruited to army service due to religious belief and practice, so the proportion of females with available information is lower than that of males (38% versus 62%). In addition, citizens may be exempted if they are religiously observant or have physical or mental disabilities. Therefore, the ultra-orthodox and disabled population may be underrepresented in this study. We believe that this underrepresentation has potential effect on the point estimates, but due to the composition of our study population, it does not alter the inspected associations.

The findings from the JPS cohort add to the knowledge regarding the long term effects of maternal prepregnancy characteristics on cardiometabolic outcomes in offspring aged 17 years, and point to populations at risk. Given the fact that in the US, 13.9% to 25.1% of births are to obese mothers [25] and the prevalence of GDM is 3%–5%, the findings of this study are important because they indicate that in the long term, these maternal characteristics have potential health consequences not only to the mother themselves but to the next generation as well. Therefore, we encourage continuous public health interventions to prevent not only GDM but also high prepregnancy mBMI, since prepregnancy maternal obesity might be the major component account for the association of GDM with the long-term health consequences of the offspring.

## Figures and Tables

**Table 1:** Characteristics of the study population.

*JPS entire cohort*, 1964–1976	GDM (*n* = 293)	No-recorded-GDM (*n* = 59499)
Maternal age at delivery (years, mean ± SD)	31.2 ± 5.9	27.6 ± 5.5
Paternal age at delivery (years, mean ± SD)	35.0 ± 7.1	31.5 ± 6.6
Birth order (mean ± SD)	1.93 ± 1.1	1.87 ± 1.1
Birth Weight (grams, mean ± SD)	3411 ± 616	3301 ± 483
Male	3495 ± 633	3347 ± 490
Female	3284 ± 569	3226 ± 462
Birth place of mother's father (%):		
Israel	10.6	12.9
West Asia	28.0	31.7
North Africa	21.2	24.4
Europe/America	40.3	31.0
Maternal education (%):		
Unknown	0.7	5.6
0–8 years	29.4	31.7
9–12 years	31.7	35.8
13+ years	38.2	26.8
Socio economic status (%)		
High	36.9	33.1
Middle	45.4	41.3
Low	17.7	25.6
Maternal health condition (%)		
Pre-eclampsia	14.4	3.4

*JPS subcohort* 1974–1976	(*n* = 77)	(*n* = 11335)
Prepregnancy maternal BMI (mean ± SD)	25.3 ± 4.4	21.9 ± 3.0
Weight gain during pregnancy (mean ± SD)	12.3 ± 6.1	11.5 ± 4.4

**Table 2:** Gender standardized anthropometry and blood pressure measured in offspring at age 17 by maternal health characteristic (*n* = 60191).

	GDM	No-recorded-GDM
Weight (kg)	69.13 ± 13.26^a^	64.05 ± 10.70
BMI (kg/m^2^)	22.47 ± 3.86^a^	21.18 ± 3.11
Systolic BP (mm HG)	121.56 ± 12.30^a^	119.84 ± 12.06
Diastolic BP (mm HG)	75.12 ± 7.44^a^	73.47 ± 8.30

^a^Significantly different, *P* < 0.05.

**Table 3:** Predictors of offspring's BMI and systolic and diastolic BP at age 17: estimated coefficients from multiple linear regression models.

*BMI at age 17*	Model 1		Model 2		Model 3	
	*β*	95%CI	*β*	95%CI	*β*	95%CI
1964–76^b^						
GDM	1.220^a^	0.863, 1.576	1.169^a^	0.814, 1.523		
Birth weight			0.586^a^	0.534, 0.638		

1974–1976^c^ subcohort						
GDM	0.950^a^	0.172, 1.728	0.719	−0.058, 1.496	0.013	−0.740 0.766
Birth weight			0.651^a^	0.502, 0.799	0.265^a^	0.116, 0.414
Prepregnancy mBMI					0.303^a^	0.281, 0.325
Weight gain in pregnancy					0.052^a^	0.038, 0.067

*Systolic BP at age 17*	*β*	95%CI	*β*	95%CI	*β*	95%CI

1964–76^b^						
GDM	1.229	−0.156, 2.613	1.206	−0.179, 2.590		
Birth weight			0.266^a^	0.063, 0.469		

1974–1976^c^ subcohort						
GDM	0.822	−1.918, 3.562	0.668	−2.058, 3.434	0.020	−2.726, 2.766
Birth weight			0.375	−0.148, 0.899	0.039	−0.503, 0.582
Prepregnancy mBMI					0.288^a^	0.207, 0.368
Weight gain in pregnancy					0.036	−0.019, 0.090

*Diastolic BP at age 17*	*β*	95%CI	*β*	95%CI	*β*	95%CI

1964–76^b^						
GDM	1.549^a^	0.587, 2.510	1.520^a^	0.559, 2.481		
Birth weight			0.333^a^	0.193, 0.474		

1974–1976^c^ subcohort						
GDM	1.582	−0.314, 3.478	1.538	−0.363, 3.439	1.167	−0.735, 3.070
Birth weight			0.123	−0.240, 0.485	−0.084	−0.459, 0.292
Prepregnancy mBMI					0.159^a^	0.103, 0.215
Weight gain in pregnancy					0.029	−0.008, 0.067

(Birth weight and weight gain in pregnancy are presented in kg).

^a^ *P* < 0.05.

^b^Model was also adjusted for: maternal age at birth, birth order, maternal ethnic origin, education, SES, maternal pre-eclampsia, and offspring gender.

^c^Model was also adjusted for: maternal age at birth, birth order, maternal ethnic origin, education, SES, maternal pre-eclampsia, offspring gender, gestational age at delivery and maternal smoking.

## Abbreviations

GDM: Gestational diabetes mellitus
JPS: Jerusalem Perinatal Study
mBMI: Maternal BMI
BP: Blood pressure.
